# Clonazepam as Agonist Substitution Treatment for Benzodiazepine Dependence: A Case Report

**DOI:** 10.1155/2013/367594

**Published:** 2013-01-30

**Authors:** Angelo Giovanni Icro Maremmani, Luca Rovai, Fabio Rugani, Silvia Bacciardi, Matteo Pacini, Liliana Dell'Osso, Icro Maremmani

**Affiliations:** ^1^Vincent P. Dole Dual Diagnosis Unit, Department of Neurosciences, Santa Chiara University Hospital, University of Pisa, Via Roma, 67, 56100 Pisa, Italy; ^2^Association for the Application of Neuroscientific Knowledge to Social Aims (AU-CNS), Lucca, 55045 Pietrasanta, Italy; ^3^G. De Lisio Institute of Behavioural Sciences, 56100 Pisa, Italy

## Abstract

Nowadays, the misuse of benzodiazepines (BZDs) is a cause for a serious concern among pharmacologically inexperienced patients, whether treated or untreated, that could lead to significant complications, including tolerance, dependence, and addiction. We present a case report in which an Italian patient affected by anxiety disorder and treated with BZDs presented a severe case of dependence on BZDs. We treated him according to an agonist substitution approach, switching from the abused BZD to a slow-onset, long-acting, high potency agonist (clonazepam), and looking at the methadone treatment model as paradigm. We decided to use clonazepam for its pharmacokinetic properties. The advantage of choosing a slow-onset, long-lasting BZD for the treatment of our patient was that it led us to a remarkable improvement in the clinical situation, including the cessation of craving, absence of withdrawal symptoms, reduced anxiety, improvements in social functioning, and a better cognition level.

## 1. Introduction

Benzodiazepines (BZDs) are prescribed in the medical management of anxiety, insomnia, seizures, and muscle spasms, but, in some patients, it can lead to significant complications, including misuse, abuse, tolerance, dependence, and addiction [1–4]. Because of these dangers, prescribing BZDs is debatable, especially in patients with severe mental illness, particularly those affected by mood, anxiety disorder [3–6], and substance use disorder, such as heroin addicts and/or alcoholics [7–9]. Indeed, the misuse of BZDs is widespread among multidrug users in the club scene; these are subjects who also exhibit high levels of other health and social problems [10]. Data from animal models which focus on the cellular and molecular basis that might underlie the addictive properties of BZDs reveal how benzodiazepines, by acting through specific GABA_(A)_ receptor subtypes, activate midbrain dopamine neurons, and how this could hijack the mesolimbic reward system [11].

Due to the possibility of abuse [12], many physicians are reluctant to prescribe BZDs [13]. Most guidance recommends that BZDs should be prescribed only for short periods and only in a minority of patients. Even so, evidence from pharmacoepidemiological studies and prescribing practice surveys show that some doctors still prescribe for longer periods and to a large number of patients [6]. Long-term benzodiazepine intoxication produces a variety of side effects, including sedation, anterograde amnesia, impaired visuospatial and visuomotor abilities, difficulties in motor coordination, psychomotor speed, and cognitive effects such as a lower speed of information processing, verbal learning and concentration and delayed response, all of which are primarily related to the dose and duration of the intake [14, 15]. When chronically prescribed benzodiazepines are discontinued, a predictable pattern of discontinuation symptoms may develop, indicating physiological dependence. Physiological dependence on benzodiazepines has been reliably described for standard and overstandard therapeutic doses, and it has been reported that withdrawal symptoms are more severe for the benzodiazepines that have a shorter elimination half-life [16, 17]. 

BZDs show differences in pharmacodynamic and pharmacokinetic profiles that explain their different therapeutic properties. There are BZDs with fast-acting anxiolytic properties and other BZDs which can act as long-acting anticonvulsants. Their different kinetic properties can be used as an indicator of their potential for abuse [18–20]. In fact, short half-life BZDs, for instance alprazolam, may lead to a greater incidence of withdrawal symptoms and addictive properties [21, 22].

To date, there is no specific or internationally recognized treatment for dependence on BZDs. While the types of intervention differ, the common aim of treatment continues to be total abstinence from benzodiazepines. There is a great deal of evidence for the superiority of agonist treatments (methadone, buprenorphine) over a withdrawal approach in opioid-dependent populations, but little research has been done on the same approach for the treatment of high-dose dependence on BZDs. However, most patients suffering from high-dose dependence on BZDs fail to achieve long-term abstinence, and in such cases some clinicians have been using BZD “substitution” treatment for decades [23]. 

In the treatment of addictive diseases, using opioid dependence as a paradigm, we know that effective treatment is based on two important pharmacological concepts: the drug used has to implement (1) an “antagonist” effect against the abused substance (blocking effect) and (2) an “anti-craving” effect against drug-seeking behaviour, by stimulating the abused substance-related system [24]. These two concepts stand as the basis of methadone treatment [25]. 

The methadone maintenance treatment (MMT) programme consists of four successive phases: induction, stabilization, maintenance, and medication withdrawal [26]. In the “induction phase,” there should be two main aims, which can be differentiated in chronological order: (1) to extinguish withdrawal symptoms at treatment entrance, by a dosage which depends on the patient's current level of acquired tolerance to opiates, and (2) to increase the dosage up to a value which ensures a narcotic blockade. Once blocking dosages have been reached, treatment proceeds with the aim of extinguishing addictive behaviours by breaking off heroin use (“stabilization phase”). The MMT philosophy is centred on the goal of its maintenance phase, which is to preserve stabilization. The philosophy of maintenance comprises two principles, the first static and the second dynamic. The static principle is to continue using the combination of therapeutic elements that have led addictive symptoms to extinction, and have allowed the achievement of a satisfactory level of personal and social functioning. Social rehabilitation is the dynamic aspect (“maintenance phase”). “Medically supervised withdrawal phase”: this is the conclusive phase of a methadone maintenance treatment programme. Withdrawal from methadone may be accomplished through a variable degree of tapering and by using various different time terms. When tapering is applied quite slowly, no withdrawal-related discomfort is reported. When, on the other hand, tapering starts after a maintenance phase with no recent dose reduction, discomfort of varying degrees may develop, depending on how steep the tapering is [26].

In this paper, we present a case in which an Italian patient affected by anxiety disorder (he had received panic disorder and social phobia diagnoses according to DSM-IV TR.) was treated with antidepressants (clomipramine) and benzodiazepines (lormetazepam) prescribed by a general practitioner. This patient showed good compliance with the treatment, but, unfortunately, his anxiety did not recede and he autonomously started to increase benzodiazepine consumption, so developing a clinical condition of misuse abuse. He was unable to stop increasing BZD intake, and he presented an enhancement of the original anxiety disorder associated with the chronic effects of using BZDs. We started with a substitution approach that switched the patient from the abused BZD to a slow-onset, long-acting, high potency BZD agonist (clonazepam). As the dosage of the abused benzodiazepine was progressively lowered the clonazepam dosage was progressively raised until the substitution was complete. In this way the patient stopped his primary abuse of BZD without any switch from intoxication to withdrawal states. His anxiety went into remission, after which the patient showed “normalized” behaviours. We supposed that these changes were due to the pharmacokinetic properties of clonazepam, in the same way seen during substitution from heroin to methadone and/or buprenorphine.

## 2. Case Presentation 

### 2.1. Anamnestic Overview

Mr. G. is a Caucasian, 52-year-old single male, now living with his family, who had requested treatment for his current benzodiazepine abuse dependence at the Vincent P. Dole Dual Diagnosis Unit, Department of Psychiatry, Santa Chiara University Hospital, Pisa (Italy). At initial psychiatric evaluation, the severity of his dependence was high, and he was also affected by a major depressive episode. After a complete diagnostic evaluation, he received a lifetime diagnosis of bipolar II disorder, benzodiazepine dependence and social anxiety disorder according to DSM-IV TR criteria [27]. 

When he was 27 years old, he presented fear and enhancement of anxiety during social events, especially when exposed to unfamiliar people. Even if he recognized that this fear was unreasonable, he was unable to control it and started to avoid all of these situations with a worsening in social and global functioning. He consulted a specialist and received a prescription of benzodiazepine (lormetazepam). Soon after starting treatment, G. reported a rapid psychopathological improvement, with lower anxiety and better social functioning. After a few months of treatment, he developed tolerance to the BZDs, with a consequent need to increase dosages. Over time, he realized that he was losing control over his BZD consumption and, progressively, anxiety was reappearing. In years, he tried a succession of psychiatrists and various different psychoactive treatments (e.g., antidepressants, mood stabilizers), but he was unable to stop or even to diminish his BZD daily dose. In addition, his clinical presentation becomes complicated by the presence of insomnia, irritability, dysphoria, poor memory, and concentration, with an impairment of learning processes and decision-making processes. At treatment entry, he presented depressed mood and a severe state of chronic benzodiazepines intoxication. He was taking lormetazepam (1 flacon/daily; 50 mg/daily) and stable low dosages of tricyclic antidepressants (trimipramine 50 mg, single dose at bedtime) and mood stabilizers (valproic acid, 300 mg twice a day).

### 2.2. Treatment Procedure

We suggested to Mr. G. the adoption of a substitution approach very similar to a methadone maintenance treatment programme. Thus, we divided intervention up into four successive phases: induction, stabilization, maintenance, and medication withdrawal [25, 26]. Supportive counselling was applied and we maintained stable dose of trimipramine and valproic acid.

#### 2.2.1. Induction Phase

First of all, we converted a half dosage of lormetazepam into a corresponding dosage of clonazepam (5 mg). We then prescribed clonazepam and lormetazepam b.i.d. After 2 weeks, we further reduced the dose of lormetazepam consumed by half, converting the fall in the dosage of lormetazepam into a corresponding increase in clonazepam. During the induction phase, Mr. G. never showed any withdrawal symptoms.

#### 2.2.2. Stabilization Phase

After 1 month of treatment, Mr. G. showed a strong improvement as regards his anxiety and stopped the self-administration of lormetazepam. 2 weeks later, we completed the substitution, leaving the patient with clonazepam as his only source of BZDs. 

#### 2.2.3. Maintenance Phase

Mr. G. felt progressively better. Neither irritability nor dysphoria was present; his hypnotic pattern gradually became normalized, and he no longer presented craving for BZDs or any abuse or binge episodes. He felt an improvement too on the cognitive level. At this stage, Mr. G. continued his regular intake of clonazepam, and he maintained a state of psychopathological well-being, with a recovery in global functioning (DSM-IV-TR: GAF range of 81–90; baseline GAF 31–40 ranged). The patient could be described as follows: minimal symptoms, good functioning in all areas, interested and involved in a wide range of activities, socially effective, and generally satisfied with life, no more than everyday problems or concerns [27].

#### 2.2.4. Medication Withdrawal Phase

After 6 months of treatment with a fixed clonazepam dosage, Mr. G. asked us to discontinue the treatment, and we decided to start the last phase. Thanks to its pharmacokinetics, clonazepam allowed us an easier discontinuation that showed fewer withdrawal symptoms. What we suggest is applying the “step by step” model, whereby the dosage of clonazepam is gradually lowered (e.g., decreasing the dose by 1–3 drops each week), so shaping a kind of homeostatic variation in the ongoing level of BZD that leaves this change “unperceived” by the brain. We reduced the daily consumption of clonazepam to the minimum level (1 mg/daily) without the recurrence of clinical symptomatology. We spent four months in this phase. 

## 3. Final Remarks

In cases of BZD dependence, there is no immediate availability of an effective tool for treatment, such as methadone in cases of opioid dependence. We are in agreement with Liebrenz and colleagues, who suggest the possibility of a maintenance treatment with a slow-onset, long-acting BZD medication as a viable option for patients who have been unable to withdraw from a problematic BZD use. We have also tried to address some of the key questions left open by Liebrenz's et al. previous work [23].

*Is there really an improvement if patients are treated with a substitution approach*?

This is highly probable. We believe that the key to obtaining the best therapeutic effects is a “stabilization” approach in which the brain system, after becoming imbalanced by the continuous “ups and downs” caused by BZD use, may be able to recover. Moreover, the advantages brought by a slow-onset, long-lasting, high potency BZD may include an improvement in health, no craving or withdrawal complications, absence of anxiety, increased compliance, improvements in social functioning, and less illegal activity, as happened with our patient.

*What is the adequate benzodiazepine for substitution*?

Clonazepam, because of its pharmacological profile—slow onset of action, half-life of 18–50 hours, high potency, and lack of active metabolites [28]—is one of the best choices among benzodiazepines. Thanks to its high potency, we were able to switch from a short to a long half-life BZD without inducing the onset of a withdrawal syndrome or reducing the amount of medication being taken. 

*Is there a clinically meaningful cognitive improvement if patients are switched from fast-onset, short-acting (e.g., flunitrazepam) to slow-onset, long-acting benzodiazepines*?

Yes. The case we presented produced very strong improvements in anxiety, hypnotic pattern, social adjustment and quality of life, and a great improvement in cognitive impairment. 

Of course, future studies on substantial samples are needed to support these observations. 
